# Interaction networks among miRNA, protein, and metabolite fingerprints identify the regulatory networks and key players in the pathogenesis of diabetic cardiomyopathy

**DOI:** 10.3389/fcell.2025.1602320

**Published:** 2025-06-30

**Authors:** Bhaswati Chatterjee, Suman S. Thakur

**Affiliations:** ^1^ National Institute of Animal Biotechnology (NIAB), Hyderabad, India; ^2^ Proteomics and Cell Signaling, Centre for Cellular and Molecular Biology, Hyderabad, India

**Keywords:** miRNAs, proteins, metabolites, protein–metabolite interaction, miRNA–protein interaction, miRNA–protein–metabolite interaction network

## Abstract

Diabetic cardiomyopathy (DCM) is a complication of diabetes and is the main cause of death in diabetic patients. The regulatory networks and key players involved in the pathogenesis of diabetic cardiomyopathy are not clearly known. We selected the miRNA, protein, and metabolite fingerprints that play a significant role in DCM and manually constructed miRNA–protein–metabolite interaction networks from the miRNA–protein and protein–metabolite interaction networks. Furthermore, protein–protein, metabolite–metabolite, and protein–metabolite interaction networks were also constructed. The miRNA–protein interaction included evidence from TarBase and microarrays/HITS-CLIP. The protein–protein, metabolite–metabolite, and protein–metabolite interaction networks were obtained at high confidence scores (≥0.7 or 70%). We proposed that the miRNA–protein–metabolite interaction networks along with their intra- and inter-connected protein–protein, metabolite–metabolite, and protein–metabolite interaction networks formed by miRNA, protein, and metabolite fingerprints such as hsa-mir-122-5p, hsa-mir-30c-5p, hsa-mir-30d-5p, hsa-mir-22-3p, IL6, GSTM2, GPX3, ACADM, GSTM3, LEP, ADIPOQ, INS, CASP1, NLRP3, HADH, ACAT1, PRDX2, PRDX1, TNF, ELAVL1, SERPINA1, A2M, IGFBP7, PRDX6, APOA1, APCS, NPPA, ADAM9, GDF15, ACADVL, ECH1, FGL1, bilirubin, butyric acid (butyrate), octanoylcarnitine (octanoylcarnit.), isoleucine, leucine, alanine, glutamine, L-valine, cytidine triphosphate (ara-CTP), 7-keto-8-aminopelargonic acid (7-keto-8-amino.), creatinine, decanoylcarnitine (decanoylcarnit.), and hexanoylcarnitine (hexanoylcarnit.) are the key players and regulatory networks involved in the pathogenesis of DCM. Notably, we also proposed that the interaction networks formed by miRNA, protein, and metabolite fingerprints involved in the early stage of DCM, such as hsa-mir-122-5p, IL6, FGL1, LEP, ADIPOQ, INS, TNF, IGFBP7, GDF15, GPX3, NPPA, bilirubin, butyric acid (butyrate), and creatinine, are the potential biomarkers and therapeutic targets for the early stage of DCM. To the best of our knowledge, this is the first study of the construction of miRNA–protein–metabolite interactomes in DCM, providing insights into the pathogenesis of DCM.

## 1 Introduction

Diabetes and heart disease share common risk factors including hypertension, dyslipidemia, obesity, and a sedentary lifestyle. Notably, insulin resistance, inflammation, and oxidative stress are responsible for the relationship between diabetes and heart disease (Suman et al., 2023). The basis of obesity prevention and treatment involves physical activity and diet besides incretin-based therapies (Ibrahim et al., 2025).

Diabetic cardiomyopathy (DCM) is a complication of diabetes, which affects 12% of diabetic patients (Kumric et al., 2021), leading to an increased risk of heart failure and death. Heart failure occurs at a later stage of DCM, while it is reversible in its early stage (Abdelrahman et al., 2021). The initial stages of DCM are mostly asymptomatic (Domínguez-Rodríguez et al., 2016). Diffuse myocardial fibrosis contributes to early DCM, as supported by the relationship between postcontrast T1 values, myocardial diastolic dysfunction, and metabolic disturbance (Jellis et al., 2011).

DCM is characterized by abnormal structure and function of the heart in diabetic patients without any hypertension, valvular heart disease and coronary artery disease (Borghetti et al., 2018). DCM is characterized by myocardial fibrosis, apoptosis, diastolic dysfunction, systolic dysfunction, and cardiomyocyte hypertrophy (Abdelrahman et al., 2021; Huynh et al., 2014). Increased NADH production in DCM through fatty acid oxidation is observed, and oxidative stress is also increased.

### 1.1 Development of diabetic cardiomyopathy

DCM development involves hyperglycemia, insulin resistance, dyslipidemia, calcium overload, protein kinase C signaling pathway activation, myocardial lipomatosis, oxidative stress, low-grade inflammation of the myocardium, and mitochondrial dysfunction (Li et al., 2019; Avagimyan et al., 2024). Pyroptosis is also related to the occurrence and development of diabetic cardiomyopathy (Geng et al., 2024).

There are four main mechanisms involved in DCM, namely, oxidative stress with the involvement of reactive oxygen species (ROS), reduced myocardial perfusion, autonomic dysfunction, and impaired glucose levels owing to insulin resistance, which leads to development of clinical heart failure (Tayanloo-Beik et al., 2021).

An increase in the triglyceride–glucose index (TyG) is a new evaluator for assessing insulin resistance and increased cardiometabolic risk. The TyG index is calculated as a logarithm [triglycerides (mmol/L)*glucose level (mg/dL)/2] (Avagimyan et al., 2025).

Notably, a type 2 diabetes mouse model can be used for development of new therapeutic strategies and translational research as they develop multiple complications simultaneously, including cardiomyopathy, neuropathy, nephropathy, liver steatosis and fibrosis, osteoporosis, and oral cavity diseases, and these complications are interconnected by hyperglycemia, insulin resistance, obesity, and systemic inflammation (Cifuentes-Mendiola et al., 2023).

### 1.2 Stages of diabetic cardiomyopathy

DCM is divided into three major stages. The early stage is usually asymptomatic and is characterized by hypertrophic heart and diastolic dysfunction with normal ejection fraction, whereas the middle stage is characterized by an increase in the size of the left ventricle, diastolic dysfunction, and a slight decrease in systolic function with an ejection fraction less than 50% (Chavali et al., 2013). The cellular mechanism of the early stage of DCM involves elevated levels of free fatty acids, changes in Ca^2+^ homeostasis, reduced expressions of GLUT-1 and GLUT-4, while the middle stage of DCM involves insulin resistance, apoptosis, necrosis, fibrosis, mild cardiovascular autonomic neuropathy, formation of advanced glycation end products, elevated levels of the renin–angiotensin–aldosterone system (RAAS), and TGF-β1, with a decrease in IGF-1 (Chavali et al., 2013). The late stage is characterized by an increase in the size of the left ventricle, impairment of both systolic and diastolic functions, and decreased heart performance, leading to heart failure (Chavali et al., 2013).

### 1.3 Diagnosis of DCM

DCM remains under-diagnosed and is not properly treated (Asghar et al., 2009). DCM is generally diagnosed with the occurrence of cardiac dysfunction (Deng et al., 2023). Echocardiography-based methods are used currently for DCM diagnosis (Maya and Villarreal, 2010). In addition, cardiac magnetic resonance imaging holds promise in detecting early stages of DCM (Tillquist and Maddox, 2012). However, these methods help detect changes in the structure and function of the heart, excluding the other reasons for the disorder (Seferović and Paulus, 2015; Murtaza et al., 2019; Tan et al., 2020; Lorenzo-Almorós et al., 2017). In addition, routine echocardiography is uneconomical and not enough sensitive to detect subclinical and asymptomatic heart damage (Pofi et al., 2021). Furthermore, no imaging techniques provide an early diagnosis of DCM (Copier et al., 2017). As DCM negatively affects the quality of life of the patient, it is important to have an early diagnosis of DCM (Zheng et al., 2021). The diagnosis of the early stage of DCM is challenging as it is asymptomatic, reversible, and also there is lack of effective biomarkers (Abdelrahman et al., 2021; Domínguez-Rodríguez et al., 2016; Seferović and Paulus, 2015; Xiong et al., 2024). Therefore, it is important to find the important players for early diagnosis of DCM, which can help in its detection before the onset of irreversible complications.

### 1.4 Interaction networks

The interaction networks between proteins and other molecular constituents including DNA, RNA, and metabolites are well coordinated in cellular functions (Liu and Gao, 2023). miRNAomics, proteomics, and metabolomics may help analyze all the miRNAs, proteins, and metabolites quantitatively in the DCM and may elucidate the miRNA, protein, and metabolite changes, respectively, in DCM. Notably, miRNAs regulate the protein-coding gene expression by interacting with target mRNAs (miRNA–mRNA interaction), and therefore to understand the specific miRNA function, it is important to map its targets (Sekar et al., 2024). Thus, apart from the experimental way of detecting the miRNA–target interaction, computational methods involving the prediction of the target also exist (Sekar et al., 2024; Bartel, 2009; Agarwal et al., 2015; Riolo et al., 2020). The computational methods detect the sequence motifs that mediate miRNA binding. The predicted miRNA binding sites are potential functional miRNA target sites, provided they are evolutionarily conserved (Sekar et al., 2024). The mediation of the function of miRNAs is through base pairing with their target (Schäfer and Ciaudo, 2020).

There are more complications involved in studying protein–metabolite interactions as it involves both proteomics and metabolomics techniques. The protein–metabolite interaction plays an important role in the regulation of protein functions, controlling various cellular processes, and this interaction works normally during healthy conditions and distorted during disease conditions (Kurbatov et al., 2023; Zhao et al., 2021).

### 1.5 Purpose of the study

Despite the clinical observations associated with DCM, the regulatory networks and key players involved in the pathogenesis of this disease are poorly understood. The microRNAs (miRNAs), proteins, and metabolites in DCM may have complementary roles by jointly performing specific biological functions such as regulation of the functions and controlling different cellular processes and the formation of DCM. These complementary roles and synergistic interactions are captured by the interaction network studies. This study aimed to build miRNA–protein–metabolite interactomes in DCM that included miRNA, protein, and metabolite fingerprints of DCM patients so as to identify the regulatory networks and key players involved in the pathogenesis of this disease.

## 2 MicroRNA, protein, and metabolite fingerprints of diabetic cardiomyopathy patients

We have selected miRNA, protein, and metabolite fingerprints of DCM patients from literature studies using PubMed.gov/Google. The miRNA, protein, and metabolite fingerprints of DCM patients were chosen based on being the biomarkers/potential biomarkers of DCM/predictors of DCM risk/play an important role in DCM and based on their differential expression in DCM with a significant decrease or increase in the miRNA/protein/metabolite levels in DCM, including their ROC curve-based biomarker analyses, other statistical methods, and different modes of measurements, summarized in Tables 1–3.

**TABLE 1:** The summary of microRNA fingerprints of diabetic cardiomyopathy.

S.No	MicroRNAs (miRNAs)	Sample	No. of patients/participants and sex/gender (male/female)	Age of patients/participants (years)	Country/place	Level	Mode of measurement	References
1	miR-1 and miR-133a	Serum	No. of patients with uncomplicated type 2 diabetes: 78 No. of controls: 12 Gender in the uncomplicated type 2 diabetes group: 78 men Gender in the control group: 12 men	Uncomplicated type 2 diabetes group: 45–65 years; control group: 45–65 years	The Netherlands	Increased	Quantitative real-time polymerase chain reaction (qPCR)-RT-qPCR	de Gonzalo-Calvo et al. (2017)
2	miR-9	Immortalized human cardiomyocytes/heart tissue	----	----	Houston	Decreased	Quantitative RT-PCR	Jeyabal et al. (2016)
3	miR-34b, miR-34c, miR-199b, miR-210, miR-650, and miR-223	Left ventricle cardiac biopsies/biopsies from peri-infarctual area/nonischemic, remote myocardium	No. of diabetic heart failure patients: 10 No. of nondiabetic heart failure patients: 19 No. of controls: 16 Gender in diabetic heart failure group: 9 men and 1 woman Gender in the nondiabetic-heart failure group: 18 men and 1 woman Gender in the control group: 9 men and 7 women	Diabetic heart failure group: 61.8 ± 2.3 (mean ± SE) Nondiabetic heart failure group: 60.2 ± 2.8 (mean ± SE) Control group: 57 ± 3.2 (mean ± SE)	Italy	Differentially expressed	qRT-PCR	Greco et al. (2012)
4	miR-216a	Left ventricle cardiac biopsies/biopsies from peri-infarctual area/nonischemic and remote myocardium	No. of diabetic heart failure patients: 10 No. of nondiabetic heart failure patients: 19 No. of controls: 16 Gender in diabetic heart failure group: 9 men and 1 woman Gender in the nondiabetic-heart failure group: 18 men and 1 woman Gender in the control group: 9 men and 7 women	Diabetic heart failure group: 61.8 ± 2.3 (mean ± SE) Nondiabetic heart failure group: 60.2 ± 2.8 (mean ± SE) Control group: 57 ± 3.2 (mean ± SE)	Italy	Increased	qRT-PCR	Greco et al. (2012)
5	miR-122-5p	serum	No. of type 2 diabetes patients completing both the baseline and 5-year follow-up CMR assessment: 51 No. of Controls (nondiabetic): 20 Gender in the type 2 diabetes group completing both the baseline and 5-year follow-up CMR assessment: 51 men Gender in controls (nondiabetic) group: 20 men	Type 2 diabetes group completing both the baseline and 5-year follow-up CMR assessment: 60 ± 7 (baseline) and 64 ± 8 (5-year follow-up) controls (nondiabetic) group: 63 ± 9	Italy	Increased	RT-qPCR	Pofi et al. (2021)
6	miR-106b-5p, miR−144-3p, miR−186-5p, miR−22-3p, and miR−30d-5p	Serum	No. of DCM patients: 3 No. of normal people:3 miR-30d-5p in DCM patients (n = 13) and normal people (n = 7)	----	China	Downregulated	Microarray/real-time quantitative PCR (qRT-PCR)	Jiang et al. (2018a), Jiang et al. (2018b)
7	miR-516a-5p, miR−575, and miR−630	Serum	No. of DCM patients: 3 No. of normal people:3	----	China	Upregulated	Microarray/real-time quantitative PCR (qRT-PCR)	Jiang et al. (2018a), Jiang et al. (2018b)
8	miR-30c and miR-181a	Formalin-fixed paraffin-embedded cardiac tissue samples	No. of DCM: 5 No. of controls:5	---	India	Downregulation	qRT-PCR	Raut et al. (2016)
9	miR-30c-5p	Plasma	No. of controls: 28 No. of diabetes patients: 26 No. of chronic heart failure patients: 22 No. of diabetes combined with chronic heart failure patients: 15; Gender in the control group: 14 men and 14 women Gender in the Diabetes group: 9 men and 17 women Gender in the chronic heart failure group: 14 men and 8 women Gender in the diabetes combined with chronic heart failure group: 10 men and 5 women	Control group: 56.6 ± 1.46 (mean ± SEM) Diabetes group: 61.6 ± 1.85 (mean ± SEM) Chronic heart failure group: 58.0 ± 3.44 (mean ± SEM) Diabetes combined with the chronic heart failure group: 60.0 ± 3.03 (mean ± SEM)	China	Negative correlation with Glucose level	qRT-PCR	Chen et al. (2017)
10	miR-126	Heart tissue	----	---	Houston	Downregulation	Real-time PCR	Babu et al. (2016)
11	miR-21	Plasma	No. of type 2 diabetes without DCM: 49 No. of type 2 diabetes with DCM: 49 Gender in the type 2 diabetes without DCM group: 22 (44.90%) women Gender in the type 2 diabetes with DCM group: 27 (55.10%) women	Type 2 diabetes without DCM group: 53.35 ± 8.29 (mean ± SD) Type 2 diabetes with DCM group: 56.33 ± 8.19 (mean ± SD)	China	Low expression	Quantitative real-time polymerase chain reaction (qRT-PCR)	Tao et al. (2020)

**TABLE 2:** The summary of proteins fingerprints of diabetic cardiomyopathy (DCM) patients.

S.No	Protein fingerprint	Samples	No. of patients/participants and sex/gender (male/Female)	Age of patients/participants (Years)	Country/ place	Protein level	Mode of measurement	Statistical method	References
1	Annexin A2	Serum	No. of normal glucose regulation (non-diabetic): 78 No. of T2DM patients: 55 No. of DCM patents: 133 Gender in normal glucose regulation (non-diabetic) group: 35 men and 43 women Gender in T2DM group:27 men and 28 women Gender in DCM group: 83 men and 50 women	Normal glucose regulation (non-diabetic) group: 43.29 ± 12.06 T2DM group: 51.56 ± 13.09^a^ DCM group: 54.85 ± 10.00^a^	China	High	Human ELISA kit	Kolmogorov–Smirnov test, ANOVA, and Multivariate logistic regression analyses	He et al. (2023)
2	PDHA1, VDAC2, ACADM, ACADVL, ACAT1, ECH1, CKMT2, MYL2, MYOZ2, TNNT2, and TPM1	Endomyocardial biopsies	No. of DCM patients: 9 No. of idiopathic dilated cardiomyopathy patients:9 No. of normal controls: 9 Gender in DCM group: 5 men and 4 women Gender in idiopathic dilated cardiomyopathy group: 5 men and 4 women Gender in the normal control group: 6 men and 3 women	DCM group: 61 ± 13 Idiopathic dilated cardiomyopathy group: 60 ± 9 Normal controls group: 58 ± 6	Italy	Upregulated	MALDI-MS/MS	Unpaired t-test, Fisher’s exact test, and one way ANOVA with Bonferroni t-test	(Frustaci et al., 2016)
3	A2M, APCS, C3, SERPINA1, GSTM2, GSTM3, PRDX1, PRDX6, GPX3, NPPA, APOA1, 3-hydroxyacyl-CoA dehydrogenase, delta3, delta2-enoyl-CoA isomerase, hydroxyacyl-coenzyme A dehydrogenase, aldehyde dehydrogenase, and peroxiredoxin 2	Endomyocardial biopsies	No. of DCM patients: 9 No. of idiopathic dilated cardiomyopathy patients:9 No. of normal controls: 9 Gender in DCM group: 5 men and 4 women Gender in idiopathic dilated cardiomyopathy group: 5 men and 4 women Gender in normal controls group: 6 men and 3 women	DCM group: 61 ± 13 Idiopathic dilated cardiomyopathy group: 60 ± 9 normal controls group: 58 ± 6	Italy	Downregulated/low expression	MALDI-MS/MS	Unpaired t-test, Fisher’s exact test, and one way ANOVA with Bonferroni t-test	Frustaci et al. (2016)
4	AGEs, IL-6, TNF-α, and insulin	Serum	No. of diabetes mellitus with normal echocardiography patients: 34 No. of diabetes mellitus with diastolic dysfunction patients: 47 No. of diabetes mellitus with systolic dysfunction patients: 32 No. of diastolic dysfunction patients: 33 No. of controls (healthy): 31 Gender in diabetes mellitus with normal echocardiography group: 16 men and 18 women Gender in diabetes mellitus with diastolic dysfunction group: 21 men and 26 women Gender in diabetes mellitus with systolic dysfunction group: 15 men and 17 women Gender in diastolic dysfunction group: 15 men and 18 women Gender in control (healthy) group: 15 men and 16 women	Diabetes mellitus with normal echocardiography group: 54.7 ± 4.1 (mean ± SD) Diabetes mellitus with diastolic dysfunction group: 56.1 ± 5.7 (mean ± SD) Diabetes mellitus with systolic dysfunction group: 55.7 ± 8.1 (mean ± SD) diastolic dysfunction group: 53.7 ± 5.4 (mean ± SD) Controls (healthy) group: 55.9 ± 3.6 (mean ± SD)	Egypt	Elevated	ELISA	Student t-test, ANOVA, nonparametric Mann–Whitney and the Kruskal–Wallis tests, and ROC curve analyses	Abdelrahman et al. (2021)
5	Adiponectin	Serum	No. of patients:100 Gender in control group: 43% men and 57% women Gender in diastolic dysfunction without diabetes group: 50% men and 50% women Gender in Diabetes mellitus with normal diastolic function group: 52% men and 48% women Gender in diabetes mellitus with diastolic dysfunction group: 57% men and 43% women	Control group: 41.67 ± 11.79 Diastolic dysfunction without diabetes group: 44.27 ± 8.31 Diabetes mellitus with normal diastolic function group: 48 ± 11.78 Diabetes mellitus with diastolic dysfunction group: 45.69 ± 8.42	West Virginia	Reduced	ELISA	ANOVA and Tukey post hoc test	(Shaver et al., 2016)
6	TNFα, leptin, and IGFBP7	Serum	No. of patients:100 Gender in control group: 43% men and 57% women Gender in diastolic dysfunction without diabetes group: 50% men and 50% women Gender in diabetes mellitus with normal diastolic function group: 52% men and 48% women Gender in diabetes mellitus with diastolic dysfunction group: 57% men and 43% women	Control group: 41.67 ± 11.79 Diastolic dysfunction without diabetes group: 44.27 ± 8.31 Diabetes mellitus with normal diastolic function group: 48 ± 11.78 Diabetes mellitus with diastolic dysfunction group: 45.69 ± 8.42	West Virginia	elevated	ELISA	ANOVA and Tukey post hoc test	(Shaver et al., 2016)
7	GDF-15	Serum	No. of patients:213 No. of patients having type 2 diabetes mellitus with diabetic cardiomyopathy: 45 No. of patients having type 2 diabetes mellitus without diabetic cardiomyopathy: 168 Gender in Type 2 diabetes mellitus with diabetic cardiomyopathy group: 29 (64%) men Gender in type 2 diabetes mellitus without diabetic cardiomyopathy group:111 (66%) men	Type 2 diabetes mellitus with diabetic cardiomyopathy group: 61.5 ± 6 Type 2 diabetes mellitus without diabetic cardiomyopathy group: 61.6 ± 6.4	Spain	Increased	ELISA	Kolmogorov–Smirnov test, binary logistic regression, and multivariate regression analysis	(Dominguez-Rodriguez et al., 2014)
8	Caspase-1, ELAVL1, and NLRP3	Heart tissue/immortalized human cardiomyocytes	----	-----	Houston	Increased	Dual-luciferase reporter assay/Western blot/immunohistochemistry/immunofluorescence	Student’s “t”-test/one-way analysis of variance, and Tukey’s multiple comparison test	(Jeyabal et al., 2016)
9	ADAM9	Heart tissue	----	----	Houston	increased	Immunohistochemistry	Mann–Whitney’s tests	Babu et al. (2016)
10	FGL-1	Serum	No. of healthy control: 58 No. of T2DM patients: 58 No. of DCM patients: 55 Gender in healthy control: 23 men/35 women Gender in T2DM patients: 28 men/30 women Gender in DCM patients: 28 men/27 women	Healthy control group: 70.97 ± 14.11 T2DM patient group: 69.76 ± 15.29 DCM patient group: 71.29 ± 13.59	China	Higher	Enzyme-linked immunosorbent assay kit	One-way analysis of variance Multivariate linear regression analyses and ROC curve	Liu et al. (2024)

Type 2 diabetes mellitus (T2DM).

^a^ P < 0.05 compared with NGR; non-diabetic subjects (NGR).

**TABLE 3:** The summary of metabolite fingerprints of diabetic cardiomyopathy patients.

S No.	Metabolite fingerprints	Sample/ specimen	No. of patients/participants and sex/gender (male/female)	Age of patients/participants (years)	Country/ place	Level of metabolites	Mode of measurement	Statistical methods	References
1	Myristoylcarnitine, lauroylcarnitine, tetradecanoyldiacylcarnitine, 3-hydroxyl-tetradecanoylcarnitine, arachidic carnitine, octadecanoylcarnitine, 3-hydroxypalmitoleylcarnitine, octanoylcarnitine, hexanoylcarnitine, and decanoylcarnitine	Plasma	No. of participants: 100 No. of patients 100 (DCM:50 patients; simple type 2 diabetes mellitus:50 patients) DCM Group: 22 Men Simple type 2 diabetes mellitus Group: 29 men	DCM group: 47.42 ± 7.43 Simple type 2 diabetes mellitus Group: 44.08 ± 5.32	China	Increase	Tandem mass spectrometer	Chi-square test/Fisher’s exact test, one-way ANOVA, Multivariable binary logistic regression, cluster heat map, and principal component analysis	(Zheng et al., 2021)
2	7-Keto-8-aminopelargonic acid, 1-piperidin-2-yl)propan-1-one, δ-undecalactone, Glu-Pro-Gly-Tyr-Ser, Indole-3-lactic acid, Ile-Phe-Val-Lys, 3-hydroxyethylchlorophyllide a, 3-devinyl-3-(1-hydroxyethyl)chlorophyllide a, Methohexital	plasma/serum	No. of DCM patients with type 2 diabetes and myocardial diastolic dysfunction: 39 No. of type 2 diabetes patients without myocardial diastolic dysfunction: 39 Male-to-female ratio is 2:1	DCM patients with type 2 diabetes and myocardial diastolic dysfunction Group: 59.41 ± 1.74 Type 2 diabetes patients without myocardial diastolic dysfunction group: 46.49 ± 1.63	China	Upregulated	UPLC-MS/MS	univariate analyses and multivariate analyses, Student’s t-test and variance multiple analysis, principal component analysis (PCA), partial least squares discriminant analysis (PLS-DA), and orthogonal partial least squares discriminant analysis (OPLS-DA)	(Hao et al., 2022)
3	2-Imino-1-imidazolidineacetic acid, metyrapone, chlorothalonil, 5-Oxo-d-bilirubin, Asp-Lys-Arg-Glu-Lys, Phe-Glu-His-Asp, N6-methyladenosine, 3′,4′-methylenedioxyorobol, N-omega-hydroxy-L-arginine, 4-Hydroxy-3-(3-methylbut-2-en-1-yl)benzoic acid, linarin, O-desmethylmycophenolic acid, 3b-(1-pyrrolidinyl)-5α-pregnane-11,20-dione, leucoside, D-glutamine, (S)-2-hydroxyglutaric acid, and 5-hydroxymethyl-2-furancarboxylic acid	plasma/serum	No. of DCM patients with type 2 diabetes and myocardial diastolic dysfunction: 39 No. of type 2 diabetes patients without myocardial diastolic dysfunction: 39 Male-to-female ratio is 2:1	DCM patients with type 2 diabetes and myocardial diastolic dysfunction group: 59.41 ± 1.74 Type 2 diabetes patients without myocardial diastolic dysfunction group: 46.49 ± 1.63	China	Downregulated	UPLC-MS/MS	univariate analyses and multivariate analyses, Student’s t-test and variance multiple analysis, principal component analysis (PCA), partial least squares discriminant analysis (PLS-DA), and orthogonal partial least squares discriminant analysis (OPLS-DA)	(Hao et al., 2022)
4	Creatinine	Serum	No. of diabetes mellitus with normal echocardiography patients: 34 No. of diabetes mellitus with diastolic dysfunction patients: 47 No. of diabetes mellitus with systolic dysfunction patients: 32 No. of diastolic dysfunction patients: 33 No. of controls (healthy): 31 Gender in diabetes mellitus with normal echocardiography Group: 16 men and 18 women Gender in diabetes mellitus with diastolic dysfunction group: 21 men and 26 women Gender in diabetes mellitus with systolic dysfunction group: 15 men and 17 women Gender in the diastolic dysfunction group: 15 men and 18 women Gender in controls (healthy) group: 15 men and 16 women	Diabetes mellitus with Normal echocardiography group: 54.7 ± 4.1 (mean ± SD) Diabetes mellitus with diastolic dysfunction group: 56.1 ± 5.7 (mean ± SD) Diabetes mellitus with systolic dysfunction group: 55.7 ± 8.1 (mean ± SD) Diastolic dysfunction group: 53.7 ± 5.4 (mean ± SD) Controls (healthy) group: 55.9 ± 3.6 (mean ± SD)	Egypt	Elevated	Enzymatic colorimetric method	Student t-test, ANOVA, nonparametric Mann–Whitney and the Kruskal–Wallis tests, and ROC curve analyses	(Abdelrahman et al., 2021)
5	Butyric acid	Plasma	No. of T2DM patients: 105 No. of DCM patients: 92 Gender in T2DM Group: 56 men and 49 women DCM Group: 48 men and 44 women	T2DM Group: 57.50 ± 15.02 (Mean ± SD) DCM Group: 60.18 ± 12.26 (Mean ± SD)	China	Decreased	Gas chromatograph	Mann–Whitney U test, and ROC	(Guo et al., 2021)
6	Bilirubin	Serum	No. of patients:100 Control Group: 43% men and 57% women Diastolic dysfunction without diabetes Group: 50% men and 50% women Diabetes mellitus with normal diastolic function Group: 52% men and 48% women Diabetes mellitus with diastolic dysfunction Group: 57% men and 43% women	Control group: 41.67 ± 11.79 Diastolic dysfunction without diabetes Group: 44.27 ± 8.31 Diabetes mellitus with normal diastolic function Group: 48 ± 11.78 Diabetes mellitus with diastolic dysfunction Group: 45.69 ± 8.42	West Virginia	Decreased	ELISA	ANOVA, Tukey post-hoc test	(Shaver et al., 2016)
7	Isoprostane	Serum	No. of Patients:100 Control Group: 43% men and 57% women Diastolic dysfunction without diabetes Group: 50% men and 50% women Diabetes mellitus with normal diastolic function Group: 52% men and 48% women Diabetes mellitus with diastolic dysfunction Group: 57% men and 43% women	Control Group: 41.67 ± 11.79 Diastolic dysfunction without diabetes Group: 44.27 ± 8.31 Diabetes mellitus with normal diastolic function Group: 48 ± 11.78 Diabetes mellitus with diastolic dysfunction Group: 45.69 ± 8.42	West Virginia	Increased	ELISA	ANOVA, Tukey post-hoc test	(Shaver et al., 2016)
8	Leucine, isoleucine, and valine	Plasma	No. of Participants: 23 No. of Patients: 11 Insulin-dependent diabetes mellitus (IDDM) patients: 6 men and 5 women Control Group (non-diabetic): 6 men and 6 women	IDDM Patients Group: 41 ± 3 (Means ± SE) Control Group (non-diabetic): 39 ± 6 (Means ± SE)	Italy	Increased	HPLC	Two-way analysis of variance, two-tailed unpaired t-test	(Avogaro et al., 1990)
9	Alanine	Blood	No. of Participants: 23 No. of Patients: 11 Insulin-dependent diabetes mellitus (IDDM) patients: 6 men and 5 women Control Group (non-diabetic): 6 men and 6 women	IDDM Patients Group: 41 ± 3 (Means ± SE) Control Group (non-diabetic): 39 ± 6 (Means ± SE)	Italy	Decrease	Semiautomated microfluorometric enzymatic technique	Two-way analysis of variance, two-tailed unpaired t-test	(Avogaro et al., 1990)
10	Saccharopine, nervonic acid, and erucic acid	Plasma	No. of Participants: 43 No. of Patients: 27 Normal Group: 8 men and 8 women T2DM Group: 6 men and 6 women DCM Group: 9 men and 6 women	Normal Group: 46.3 ± 8.7^a^ T2DM Group: 48.5 ± 9.3 DCM Group: 52.0 ± 6.7	China	Increase	LC-MS	One-way ANOVA test, data filtering was based on the interquartile range and ROC curve	(Xiong et al., 2024)
11	11-ketoetiocholanolone, cytidine triphosphate	Plasma	No. of Participants: 43 No. of Patients: 27 Normal Group: 8 men and 8 women T2DM Group: 6 Males and 6 Females DCM Group: 9 Males and 6 Females	Normal Group: 46.3 ± 8.7^a^ T2DM Group: 48.5 ± 9.3 DCM Group: 52.0 ± 6.7	China	Decrease	LC-MS	One-way ANOVA test, data filtering was based on the interquartile range and ROC curve	(Xiong et al., 2024)

Type 2 diabetes mellitus (T2DM).

^a^ Significant differences compared to the DCM, group with *P* < 0.01.

### 2.1 MicroRNA fingerprints of diabetic cardiomyopathy patients, as obtained from the studies in different countries

The miRNA fingerprints of DCM are summarized in Table 1.

#### 2.1.1 Study from the Netherlands

In a study from the Netherlands, it was found that the levels of miR-1 and miR-133a were increased in myocardial steatosis in type 2 diabetes as they used the samples from the Pioglitazone Influence on triglyceride Accumulation in the Myocardium In Diabetes (PIRAMID) study (de Gonzalo-Calvo et al., 2017). Myocardial steatosis is an indicator of diabetic cardiomyopathy. These miRNAs can be used for the detection of subclinical diabetic cardiomyopathy (de Gonzalo-Calvo et al., 2017).

#### 2.1.2 Studies from Italy

The miR-34b, miR-34c, miR-199b, miR-210, miR-650, and miR-223 were differentially expressed between diabetic ischemic heart failure and nondiabetic ischemic heart failure patients, while miR-216a was at a higher level in both diabetic ischemic heart failure and nondiabetic ischemic heart failure patients, as per a study from Italy (Greco et al., 2012).

Interestingly, a 5-year follow-up study on T2DM patients participating in the Cardiovascular Effects of Chronic Sildenafil in Men With Type 2 Diabetes (CECSID) trial confirmed an increase in miR-122-5p in diabetic heart patients, suggesting miR-122-5p as a biomarker for subclinical diastolic dysfunction and early diabetic cardiomyopathy stage, including triggering the progression of diabetic cardiomyopathy, in a study from Italy (Pofi et al., 2021).

#### 2.1.3 Studies from China

miR-21 levels were found to be significantly lowered in DCM patients compared to non-DCM patients in a study from China, suggesting miR-21 as a biomarker for DCM diagnosis (Tao et al., 2020). Furthermore, the downregulation of miR-106b-5p, miR−144-3p, miR−186-5p, miR−22-3p, and miR−30d-5p and upregulation of miR-516a-5p, miR−575, and miR−630 were observed in DCM patients in a study from China (Jiang et al., 2018a; Jiang et al., 2018b). Notably, Huang et al. (2022) found in streptozotocin (STZ)-induced diabetic rats with type 1 diabetes that low expression levels of exosomal miR-30d-5p and miR-126a-5p were found to be linked with heart failure with preserved ejection fraction (Huang et al., 2022).

A negative correlation was observed between miR-30c-5p and glucose levels in chronic heart failure patients and chronic heart failure with diabetes patients in a study from China. The mir30c relative expression in the heart was found by qRT-PCR (Chen et al., 2017). Notably, a significant reduction in mir30c expression was found in the heart of 24-week-old db/db diabetic cardiomyopathy mice compared to control mice showing the association of Mir30c with diabetic heart failure pathogenesis (Chen et al., 2017). Interestingly, severe diabetes and cardiomyopathy, developed by 8 weeks of age in diabetic db/db mice, were found in the presence of cardiac hypertrophy (Chen et al., 2017).

#### 2.1.4 Study from India

The downregulation of miR-30c and miR-181a expressions was associated with an increase in the myocardial expression of p53 and p21, suggesting these miRNAs have a synergistic effect on p53-p21 pathways in cardiac hypertrophy induced by diabetes, as found from a study in India (Raut et al., 2016).

#### 2.1.5 Studies from Houston

A significant downregulated miR-9 expression was observed in the diabetic heart compared to the healthy non-diabetic heart in a study from Houston (Jeyabal et al., 2016). In another study, a downregulation of the expression of miR-126 was observed in diabetic patient hearts as compared to the non-diabetic patient heart tissues (Babu et al., 2016).

### 2.2 Protein fingerprints of diabetic cardiomyopathy patients, as obtained from studies in different countries

The protein fingerprints of the DCM are summarized in Table 2.

#### 2.2.1 Study from Egypt

Abdelrahman et al. (2021), found that elevated levels of advanced glycation end-products (AGEs), IL-6, TNF-α, and insulin can be used for early prediction of DCM, as observed from a study in Egypt (Abdelrahman et al., 2021).

#### 2.2.2 Study from Germany

The human diabetic heart has increased heat shock protein 27 (Hsp27) modification with methylglyoxal modification argpyrimidine and phosphorylation, showing an association between diabetes and increased Hsp27 modification (Gawlowski et al., 2009).

#### 2.2.3 Studies from Houston

Jeyabal et al. (2016), in a study from Houston, found that the human diabetic heart has increased expressions of caspase-1 and ELAVL1 compared to the non-diabetic heart. Caspase-1 involves regulation of increased movement of inflammatory cells into the diabetic heart myocardium, and the diabetic heart failure patients have also been found to have increased expression of NLRP3 (Jeyabal et al., 2016). Furthermore, it was also found that diabetic heart failure patients exhibited high levels of ADAM9 compared to non-diabetic normal heart tissues (Babu et al., 2016).

#### 2.2.4 Study in the West Virginian population

In the West Virginian Population, adiponectin levels were lower in diabetes with diastolic dysfunction patients (preclinical DCM) compared to diabetes patients and diastolic dysfunction patients, while TNF-α and leptin levels were increased in diabetes patients and diabetes with diastolic dysfunction patients compared to the controls. Furthermore, higher levels of IGFBP7 were found in diabetes with diastolic dysfunction patients (Shaver et al., 2016).

#### 2.2.5 Study from Spain

Notably, the level of growth differentiation factor-15 (GDF-15) was found to be higher in DCM patients with type 2 diabetes in a study from Spain (Dominguez-Rodriguez et al., 2014) and is thus a prognostic marker in DCM (Domínguez-Rodríguez et al., 2016).

#### 2.2.6 Studies from China

The concentration of annexin A2, which is a calcium-dependent phospholipid-binding protein, was found to be higher in the serum of DCM patients, in a study from China, and was negatively associated with systolic and diastolic functions of the heart, and this may help in early diagnosis of DCM (He et al., 2023). In another study from China, the researchers found a significant increase in serum FGL-1 in DCM patients compared to healthy and T2DM patients, suggesting the potential of FGL-1 for early diagnosis of DCM and as its therapeutic target (Liu et al., 2024).

#### 2.2.7 Study from Italy

In a study from Italy (Frustaci et al., 2016), the endomyocardial biopsies of DCM and idiopathic dilated cardiomyopathy patients were compared with surgical biopsies of patients with mitral stenosis and normal left ventricular dimensions and functions, which served as the control, and it was found that mitochondrial damage, myofibrillolysis, ROS, and apoptosis was higher in DCM compared to idiopathic dilated cardiomyopathy patients and control. Interestingly, PDHA1, VDAC2, ACADM, ACADVL, ACAT1, ECH1, CKMT2, MYL2, MYOZ2, TNNT2, and TPM1 expressions were upregulated in DCM and idiopathic dilated cardiomyopathy patients, while A2M, APCS, C3, SERPINA1, GSTM2, GSTM3, PRDX1, PRDX6, GPX3, NPPA, and APOA1 expressions were downregulated in DCM and idiopathic dilated cardiomyopathy patients. DCM patients had a low expression of 3-hydroxyacyl-CoA dehydrogenase, delta3, delta2-enoyl-CoA isomerase, and hydroxyacyl-coenzyme A dehydrogenase proteins which are involved in lipid metabolism associated along with the low expressions of aldehyde dehydrogenase and peroxiredoxin 2 (Frustaci et al., 2016).

### 2.3 Metabolite fingerprints of diabetic cardiomyopathy patients, as obtained from studies in different countries

The metabolite fingerprints of the DCM patients are summarized in Table 3.

#### 2.3.1 Studies from China

The early stages of DCM are characterized by myocardial diastolic dysfunction. Interestingly, Hao et al. (2022) found the top 20 differentially regulated metabolites, namely, 2-imino-1-imidazolidineacetic acid, metyrapone, chlorothalonil, 5-oxo-d-bilirubin, Asp-Lys-Arg-Glu-Lys, gossypol, Phe-Glu-His-Asp, N6-methyladenosine, 3′,4′-methylenedioxyorobol, N-omega-hydroxy-L-arginine, 4-hydroxy-3-(3-methylbut-2-en-1-yl)benzoic acid, linarin, O-desmethylmycophenolic acid, 3b-(1-pyrrolidinyl)-5α-pregnane-11,20-dione, leucoside, D-glutamine (S)-2-hydroxyglutaric acid, and 5-hydroxymethyl-2-furancarboxylic acid metabolites, to be downregulated, and 7-keto-8-aminopelargonic acid, 1-(piperidin-2-yl)propan-1-one, δ-undecalactone, Glu-Pro-Gly-Tyr-Ser, indole-3-lactic acid, Ile-Phe-Val-Lys, 3-hydroxyethylchlorophyllide a, 3-devinyl-3-(1-hydroxyethyl) chlorophyllide a, and methohexital metabolites were found to be upregulated in myocardial diastolic dysfunction of DCM patients with type 2 diabetes, in a study from China (Hao et al., 2022). Notably, the level of butyric acid was found to be decreased in DCM compared to T2DM patients as found in another study from China (Guo et al., 2021). DCM involves fatty acid oxidation with acylcarnitine as its intermediate product. Fatty acid oxidation is dysregulated in DCM with an accumulation of lipids in the heart, thereby causing DCM. The increased levels of medium- and long-chain acylcarnitine such as myristoylcarnitine, lauroylcarnitine, tetradecanoyldiacylcarnitine, 3-hydroxyl-tetradecanoylcarnitine, arachidic carnitine, octadecanoylcarnitine, 3-hydroxypalmitoleylcarnitine, octanoylcarnitine, hexanoylcarnitine, and decanoylcarnitine were found to be positively associated with diabetic cardiomyopathy risk in type 2 diabetes mellitus (T2DM) patients from China (Zheng et al., 2021).

Xiong et al. (2024) compared patients of T2DM with or without DCM and controls in a study from China and found by the biomarker analysis using an ROC curve with an area under the curve greater than 0.75 that cytidine triphosphate, 11-ketoetiocholanolone, saccharopine, nervonic acid, and erucic acid are potential biomarkers of DCM (Xiong et al., 2024).

#### 2.3.2 Study from Egypt

The increased level of creatinine can be used for early prediction of DCM, as found in the study from Egypt (Abdelrahman et al., 2021).

#### 2.3.3 Study in the West Virginian population

Notably, decreased bilirubin and increased levels of isoprostane were found in the diabetes with diastolic dysfunction group in the West Virginian population (Shaver et al., 2016).

#### 2.3.4 Studies from Italy

In a study from Italy, in T1DM (insulin-dependent diabetes mellitus), the human heart released less alanine and more branched-chain amino acids such as leucine, isoleucine, and valine compared to the control (healthy heart), evidencing the increased branched-chain amino acid production in the diabetic myocardium (Avogaro et al., 1990; Sowton et al., 2019).

## 3 Interaction network studies involving protein and metabolite fingerprints of diabetic cardiomyopathy patients

The protein and metabolite fingerprints of diabetic cardiomyopathy patients (Tables 2, 3) were analyzed together using the STITCH database (Szklarczyk et al., 2016) to study their interaction network at a cut-off of high confidence scores (≥0.7 or 70%). The STITCH database incorporates the details from text mining, co-occurrence, co-expression, experiments, gene fusion, neighborhood, homology, predictions, and databases.

### 3.1 Protein–protein interaction network studies between protein fingerprints of diabetic cardiomyopathy patients

**FIGURE 1:**
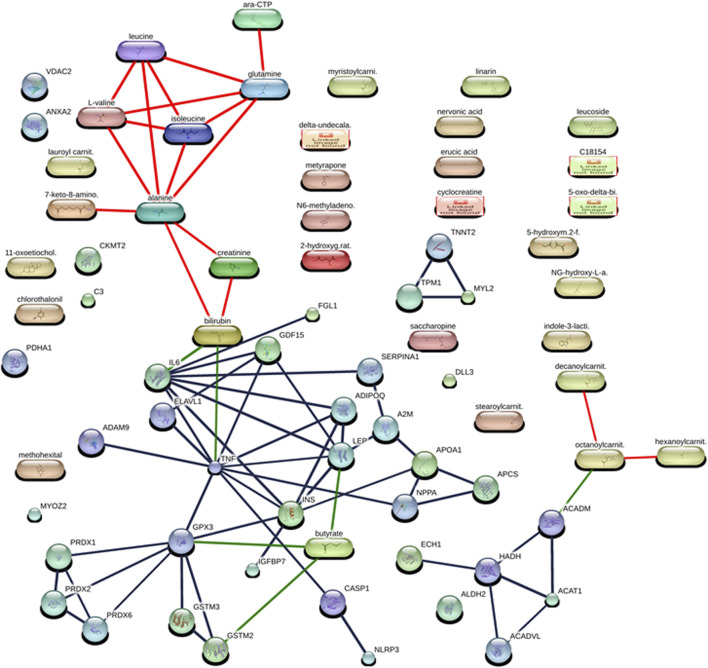
Interaction networks between protein and metabolites of diabetic cardiomyopathy (DCM) patients at the confidence view with high confidence scores (≥70%). The protein–protein interactions are represented in gray, protein–metabolite interactions are represented in green, and metabolite–metabolite interactions are represented in red.

We found that the studied protein fingerprints of DCM (Table 2) formed protein–protein interaction networks among them at high confidence scores (≥70%) by using the STITCH database (Szklarczyk et al., 2016). These protein–protein interactions were found between TNNT2 and TPM1 (having a high confidence score of 0.997 or 99.7% including the coexpression, experiment, database, and text mining-based scores), GSTM3 and GSTM2 (having a high confidence score of 0.995 or 99.5% including the homology, experiment, database, and text mining-based scores), LEP and IL6 (having a high confidence score of 0.994 or 99.4% including the database and text mining-based scores), ADIPOQ and LEP (having a high confidence score of 0.988 or 98.8% including the text mining-based scores), LEP and INS (having a high confidence score of 0.988 or 98.8% including the text mining-based scores), CASP1 and NLRP3 (having a high confidence score of 0.987 or 98.7% including the database and text mining-based scores), HADH and ACAT1 (having a high confidence score of 0.984 or 98.4% including the neighborhood on chromosome, gene fusion, coexpression, experiment, database, and text mining-based scores), ADIPOQ and INS interaction (having a high confidence score of 0.981 or 98.1% including the text mining-based scores), TNNT2 and MYL2 (having a high confidence score of 0.979 or 97.9% including the coexpression, experiment, database, text mining-based scores), PRDX2 and PRDX1 (having a high confidence score of 0.975 or 97.5% including the phylogenetic cooccurrence, homology, experiment, and text mining-based scores), IL6 and INS (having a high confidence score of 0.972 or 97.2% including the text mining-based scores), TPM1 and MYL2 interaction (having a high confidence score of 0.969 or 96.9% including the coexpression, experiment, database, and text mining-based scores), ACADM and ACAT1 (having a high confidence score of 0.966 or 96.6% including the neighborhood on chromosome, coexpression, database, and text mining-based scores), TNF and ELAVL1 (having a high confidence score of 0.963 or 96.3% including text mining-based scores), ADIPOQ and IL6 (having a high confidence score of 0.962 or 96.2% including text mining-based scores), SERPINA1 and A2M (having a high confidence score of 0.948 or 94.8% including the database and text mining-based scores), IGFBP7 and INS (having a high confidence score of 0.944 or 94.4% including the experiment and text mining-based scores), PRDX6 and PRDX2 (having a high confidence score of 0.94 or 94% including the homology, coexpression, experiment, and text mining-based scores), TNF and GPX3 (having a high confidence score of 0.933 or 93.3% including text mining-based scores), GPX3 and GSTM3 (having a high confidence score of 0.933 or 93.3% including database and text mining-based scores), A2M and APOA1 (having a high confidence score of 0.931 or 93.1% including database and text mining-based scores), GPX3 and GSTM2 (having a high confidence score of 0.93 or 93% including database and text mining-based scores), A2M and LEP (having a high confidence score of 0.918 or 91.8% including experiment and text mining-based scores), APCS and APOA1 (having a high confidence score of 0.911 or 91.1% including coexpression, database, and text mining-based scores), SERPINA1 and IL6 (having a high confidence score of 0.903 or 90.3% including text mining-based scores), NPPA and APOA1 (having a high confidence score of 0.902 or 90.2% including database and text mining-based scores), NPPA and APCS (having a high confidence score of 0.9 or 90% including database-based scores), ADAM9 and TNF (having a high confidence score of 0.895 or 89.5% including experiment and text mining-based scores), TNF and ADIPOQ (having a high confidence score of 0.887 or 88.7% including text mining-based scores), TNF and IL6 (having a high confidence score of 0.877 or 87.7% including coexpression and text mining-based scores), IL6 and GDF15 (having a high confidence score of 0.874 or 87.4% including text mining-based scores), GPX3 and INS interaction (having a high confidence score of 0.851 or 85.1% including text mining-based scores), HADH and ACADVL (having a high confidence score of 0.841 or 84.1% including neighborhood on chromosome, phylogenetic cooccurrence, coexpression, and text mining-based scores), TNF and GDF15 (having a high confidence score of 0.837 or 83.7% including text mining-based scores), TNF and NPPA (having a high confidence score of 0.831 or 83.1% including text mining-based scores), LEP and GDF15 (having a high confidence score of 0.822 or 82.2% including text mining-based scores), ELAVL1 and GDF15 (having a high confidence score of 0.822 or 82.2% including text mining-based scores), GPX3 and PRDX2 (having a high confidence score of 0.814 or 81.4% including experiment and text mining-based scores), HADH and ECH1 (having a high confidence score of 0.81 or 81% including co-expression and text mining-based scores), IL6 and FGL1 (having a high confidence score of 0.81 or 81% including text mining-based scores), CASP1 and TNF (having a high confidence score of 0.804 or 80.4% including text mining-based scores), GPX3 and PRDX1 (having a high confidence score of 0.803 or 80.3% including experiment and text mining-based scores), PRDX6 and PRDX1 (having a high confidence score of 0.791 or 79.1% including homology, co-expression, experiment, and text mining-based scores), ACADM and HADH (having a high confidence score of 0.783 or 78.3% including neighborhood on chromosome, co-expression, and text mining-based scores), TNF and INS (having a high confidence score of 0.755 or 75.5% including text mining-based scores), INS and APOA1 (having a high confidence score of 0.726 or 72.6% including text mining-based scores), TNF and LEP (high confidence score of 0.72 or 72% including text mining-based scores), ACADVL and ACAT1 (having a high confidence score of 0.709 or 70.9% including neighborhood on chromosome, co-expression, and text mining-based scores), GPX3 and PRDX6 (having a high confidence score of 0.709 or 70.9% including co-expression, experiment, and text mining-based scores) (Figure 1; Supplementary Table S1).

### 3.2 Metabolite–metabolite interaction network studies between metabolite fingerprints of diabetic cardiomyopathy patients

The studied metabolite fingerprints of DCM (Table 3) analyzed by the STITCH database (Szklarczyk et al., 2016) at high confidence scores (≥70%) showed metabolite–metabolite interaction between them, such as isoleucine–leucine interaction (having a high confidence score of 0.999 or 99.9% including the homology, database, and text mining-based scores), alanine–glutamine interaction (having a high confidence score of 0.998 or 99.8% including the homology, database, and text mining-based scores), alanine–leucine interaction (having a high confidence score of 0.996 or 99.6% including the homology, database, and text mining-based scores), glutamine–leucine interaction (having a high confidence score of 0.996 or 99.6% including the homology, database, and text mining-based scores), isoleucine–L-valine interaction (having a high confidence score of 0.992 or 99.2% including the homology, database, and text mining-based scores), alanine–isoleucine interaction (having a high confidence score of 0.991 or 99.1% including the homology, database, and text mining-based scores), leucine–L-valine interaction (having a high confidence score of 0.987 or 98.7% including the homology, database, and text mining-based scores), glutamine–isoleucine interaction (having a high confidence score of 0.982 or 98.2% including the homology, database, and text mining-based scores), alanine–L-valine interaction (having a high confidence score of 0.976 or 97.6% including the homology, database, and text mining-based scores), glutamine–L-valine interaction (having a high confidence score of 0.951 or 95.1% including the homology, database, and text mining-based scores), cytidine triphosphate (ara-CTP)–glutamine interaction (having a high confidence score of 0.95 or 95% including the database and text mining-based scores), 7-keto-8-aminopelargonic acid (7-keto-8-amino.)–alanine interaction (having a high confidence score of 0.914 or 91.4% including the database and text mining-based scores), bilirubin–creatinine interaction (having a high confidence score of 0.876 or 87.6% including the text mining-based scores), creatinine–alanine interaction (having a high confidence score of 0.859 or 85.9% including text mining-based scores), bilirubin–alanine interaction (having a high confidence score of 0.851 or 85.1% including text mining-based scores), octanoylcarnitine (octanoylcarnit.)–decanoylcarnitine (decanoylcarnit.) interaction (having a high confidence score of 0.739 or 73.9% including homology and text mining-based scores), octanoylcarnitine (octanoylcarnit.)–hexanoylcarnitine (hexanoylcarnit.) interaction (having a high confidence score of 0.739 or 73.9% including homology and text mining-based scores) (Figure 1; Supplementary Table S1).

### 3.3 Protein–metabolite interaction network studies between protein and metabolite fingerprints of diabetic cardiomyopathy (DCM) patients

The protein and metabolite fingerprints of DCM patients (Tables 2, 3) were found to form protein–metabolite interaction networks at high confidence scores (≥70%) using the STITCH database (Szklarczyk et al., 2016). These protein–metabolite interaction networks were as follows IL6–bilirubin interaction (having a high confidence score of 0.934 or 93.4% including the database and text mining-based scores), GPX3–butyric acid (butyrate) interaction (having a high confidence score of 0.853 or 85.3% including text mining-based scores), LEP–butyric acid (butyrate) interaction (having a high confidence score of 0.842 or 85.3% including text mining-based scores), GSTM2–butyric acid (butyrate) interaction (having a high confidence score of 0.824 or 82.4% including database and text mining-based scores), TNF–bilirubin interaction (having a high confidence score of 0.752 or 75.2% including text mining-based scores), ACADM–octanoylcarnitine (octanoylcarnit.) interaction (having a high confidence score of 0.75 or 75% including text mining-based scores) (Figure 1; Supplementary Table S1).

## 4 miRNA–protein interaction network studies between miRNA and protein fingerprints of diabetic cardiomyopathy patients

**FIGURE 2:**
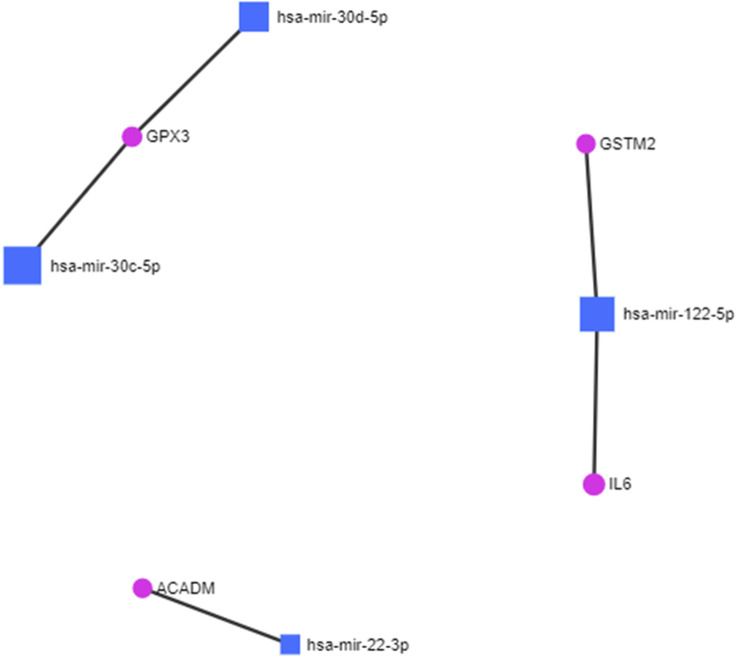
miRNA–protein interactions in diabetic cardiomyopathy.

The protein fingerprints (Table 2) forming the protein–metabolite interaction networks (Figure 1; SupplementaryTable S1) were selected for finding and studying the miRNA–protein interactions. The miRNA fingerprints (Table 1) and the selected proteins fingerprints, selected from those proteins fingerprints, forming the protein–metabolite interaction networks (Figure 1; Supplementary Table S1), were analyzed together using the miRNet web tool (miRNet, 2024; Fan and Xia, 2018; Fan et al., 2016) to study the interaction between miRNAs and proteins. The miRNet is a miRNA-centric network visual analytics platform (miRNet). The miRNA–protein interactions were found using the miRNet web tool (miRNet, 2024; Fan and Xia, 2018; Fan et al., 2016) with a degree filter cutoff of default value 1 and applying it to all network nodes. The miRNA–protein interaction networks were hsa-mir-122-5p–IL6 interaction (identified by TarBase and experiment such as microarrays), hsa-mir-122-5p–GSTM2 interaction (identified by TarBase and experiment such as microarrays), hsa-mir-30c-5p–GPX3 interaction (identified by TarBase and experiment such as HITS-CLIP), hsa-mir-30d-5p–GPX3 interaction (identified by TarBase and experiment such as HITS-CLIP), and hsa-mir-22-3p–ACADM interaction (identified by TarBase and experiment such as HITS-CLIP) (Figure 2; Supplementary Table S2) using the miRNet web tool (miRNet, 2024; Fan and Xia, 2018; Fan et al., 2016).

## 5 miRNA–protein–metabolite interactome studies in diabetic cardiomyopathy patients

In the construction of the miRNA–protein–metabolite interaction networks, we found that proteins interact with both miRNA and metabolites. Therefore, we manually integrated the miRNA–protein interaction network with the protein–metabolite interaction network through the protein, resulting in the construction of miRNA–protein–metabolite interaction networks. We found five miRNA–protein–metabolite interaction networks in DCM.

**FIGURE 3:**
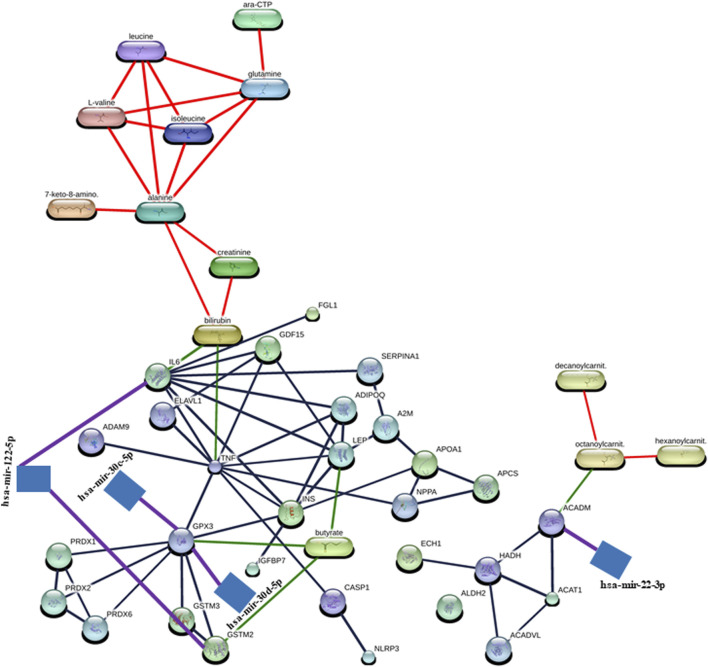
Representation of miRNA–protein–metabolite interactome. The miRNA–protein interactions are represented in purple, protein–metabolite interactions are represented in green, protein–protein interaction are represented in gray, and metabolite–metabolite interactions are represented in red.

Notably, in the construction of the hsa-mir-122-5p–IL6 – bilirubin (miRNA–protein–metabolite) interaction network (Figure 3), the hsa-mir-122-5p–IL6 (miRNA–protein) interaction network was identified by TarBase and experiment such as microarrays (Figure 2; Supplementary Table S2) using the miRNet web tool (miRNet, 2024; Fan and Xia, 2018; Fan et al., 2016). Furthermore, IL6 (protein) was found to interact with bilirubin (metabolite) with a high confidence score of 0.934 or 93.4% including the database and text mining-based scores forming the IL6–bilirubin (protein–metabolite) interaction network (Figure 1; Supplementary Table S1) using the STITCH database (Szklarczyk et al., 2016). Thus, IL6 (protein) was found to interact with both hsa-mir-122-5p (miRNA) and bilirubin (metabolite). Therefore, the hsa-mir-122-5p–IL6 – bilirubin (miRNA–protein–metabolite) interaction network (Figure 3) was constructed by manually integrating the hsa-mir-122-5p–IL6 (miRNA–protein) interaction network with IL6–bilirubin (protein–metabolite) interaction network through the protein IL6 (Figure 3).

Similarly, another miRNA–protein–metabolite interaction network such as hsa-mir-122-5p–GSTM2–butyric acid (butyrate) interaction (Figure 3) was constructed by manually integrating the miRNA–protein interaction network such as hsa-mir-122-5p–GSTM2 interaction [identified by TarBase and experiment such as microarrays using the miRNet web tool (miRNet, 2024; Fan and Xia, 2018; Fan et al., 2016) (Figure 2; Supplementary Table S2)] with the protein–metabolite interaction network such as GSTM2–butyric acid (butyrate) interaction [having a high confidence score of 0.824 or 82.4% including database and text mining-based scores using the STITCH database (Szklarczyk et al., 2016) (Figure 1; Supplementary Table S1)] through the protein GSTM2 (Figure 3).

Furthermore, another interaction network such as hsa-mir-30c-5p–GPX3 - butyric acid (butyrate) (miRNA–protein–metabolite) interaction (Figure 3) was formed by manually integrating the miRNA–protein interaction network such as hsa-mir-30c-5p–GPX3 interaction [identified by TarBase and experiment such as HITS-CLIP using the miRNet web tool (miRNet, 2024; Fan and Xia, 2018; Fan et al., 2016) (Figure 2; Supplementary Table S2)] with the protein–metabolite interaction network such as GPX3–butyric acid (butyrate) interaction [having a high confidence score of 0.853 or 85.3% including text mining-based scores using the STITCH database (Szklarczyk et al., 2016) (Figure 1; Supplementary Table S1)] through the protein GPX3 (Figure 3).

Furthermore, another miRNA–protein–metabolite interaction network such as hsa-mir-30d-5p–GPX3–butyric acid (butyrate) interaction (Figure 3) was constructed by manually integrating the hsa-mir-30d-5p–GPX3 (miRNA–protein) interaction network [identified by TarBase and experiment such as HITS-CLIP using the miRNet web tool (miRNet, 2024; Fan and Xia, 2018; Fan et al., 2016) (Figure 2; Supplementary Table S2)] with the GPX3–butyric acid (butyrate) (protein–metabolite) interaction network [(having a high confidence score of 0.853 or 85.3% including text mining-based scores using the STITCH database (Szklarczyk et al., 2016) (Figure 1; Supplementary Table S1)] through the protein GPX3 (Figure 3).

Another miRNA–protein–metabolite interaction network involving hsa-mir-22-3p–ACADM–octanoylcarnitine (octanoylcarnit.) interaction (Figure 3) resulted by manually integrating hsa-mir-22-3p–ACADM (miRNA–protein) interaction [identified by TarBase and experiment such as HITS-CLIP using the miRNet web tool (miRNet, 2024; Fan and Xia, 2018; Fan et al., 2016) (Figure 2; Supplementary Table S2)] with the ACADM–octanoylcarnitine (octanoylcarnit.) (protein–metabolite) interaction network [(having a high confidence score of 0.75 or 75% including text mining-based scores using the STITCH database (Szklarczyk et al., 2016) (Figure 1; Supplementary Table S1)] through the protein ACADM (Figure 3). Notably, octanoylcarnitine (octanoylcarnit.) is further interacting with different metabolites forming metabolite–metabolite interactions such as octanoylcarnitine (octanoylcarnit.)–decanoylcarnitine (decanoylcarnit.) interaction and octanoylcarnitine (octanoylcarnit.)–hexanoylcarnitine (hexanoylcarnit.) interaction at high confidence scores (≥0.7 or 70%) (Figure 3; Supplementary Table S1). Furthermore, ACADM is forming protein–protein interactions such as ACADM–HADH interaction and ACADM–ACAT1 interaction at high confidence scores (≥0.7 or 70%) (Figure 3; Supplementary Table S1). Furthermore, the interactions between HADH and ACAT1, HADH and ACADVL, HADH and ECH1, ACADVL and ACAT1 are found at high confidence scores (≥0.7 or 70%) (Figure 3; Supplementary Table S1).

Interestingly, butyric acid (butyrate) is further interacting with protein LEP, forming LEP–butyric acid (butyrate) interaction at high confidence scores (≥0.7 or 70%) (Figure 3; Supplementary Table S1). Furthermore, bilirubin interacts with TNF, forming TNF–bilirubin interaction at high confidence scores (≥0.7 or 70%) (Figure 3; Supplementary Table S1). TNF is also found to be interacting with various proteins, forming TNF–ELAVL1 interaction, TNF–GPX3 interaction, ADAM9–TNF interaction, TNF–ADIPOQ interaction, TNF–IL6 interaction, TNF–GDF15 interaction, TNF–NPPA interaction, CASP1–TNF interaction, TNF–INS interaction, and TNF–LEP interaction at high confidence scores (≥0.7 or 70%) (Figure 3; Supplementary Table S1). Interestingly, LEP is found to be interacting with various proteins, forming protein–protein interactions including LEP–IL6 interaction, ADIPOQ–LEP interaction, LEP–INS interaction, A2M–LEP interaction, and LEP–GDF15 interaction apart from the TNF–LEP interaction at high confidence scores (≥0.7 or 70%) (Figure 3; Supplementary Table S1). The GPX3 forms protein–protein interactions including GPX3–GSTM3 interaction, GPX3–GSTM2 interaction, GPX3–INS interaction, GPX3–PRDX2 interaction, GPX3–PRDX1 interaction, and GPX3–PRDX6 interaction apart from the TNF–GPX3 interaction at high confidence scores (≥0.7 or 70%) (Figure 3; Supplementary Table S1). The GSTM2 forms the GSTM3–GSTM2 interaction apart from the GSTM3–GSTM2 interaction at high confidence scores (≥0.7 or 70%) (Figure 3; Supplementary Table S1). The protein IL6 is found to form protein–protein interactions including IL6–INS interaction, ADIPOQ–IL6 interaction, SERPINA1–IL6 interaction, and IL6–GDF15 interaction apart from LEP–IL6, IL6–FGL1 and TNF–IL6 interactions at high confidence scores (≥0.7 or 70%) (Figure 3; Supplementary Table S1). In addition, protein–protein interactions such as CASP1–NLRP3, ADIPOQ–INS, PRDX2–PRDX1, SERPINA1–A2M, IGFBP7–INS, PRDX6–PRDX2, A2M–APOA1, APCS–APOA1, NPPA–APOA1, NPPA–APCS, ELAVL1–GDF15, PRDX6–PRDX1, and INS–APOA1 were found at high confidence scores (≥0.7 or 70%) (Figure 3; Supplementary Table S1). Additionally, metabolite-metabolite interactions were also found, such as isoleucine–leucine interaction, alanine–glutamine interaction, alanine–leucine interaction, glutamine–leucine interaction, isoleucine–L-valine interaction, alanine–isoleucine interaction, leucine–L-valine interaction, glutamine–isoleucine interaction, alanine–L-valine interaction, glutamine–L-valine interaction, cytidine triphosphate (ara-CTP)–glutamine interaction, 7-keto-8-aminopelargonic acid (7-keto-8-amino.)–alanine interaction, bilirubin–creatinine interaction, creatinine–alanine interaction, and bilirubin–alanine interaction at high confidence scores (≥0.7 or 70%) (Figure 3; Supplementary Table S1).

## 6 Conclusion

We have manually constructed miRNA–protein–metabolite interaction networks such as hsa-mir-122-5p–IL6–bilirubin, hsa-mir-122-5p–GSTM2 - butyric acid (butyrate), hsa-mir-30c-5p–GPX3 - butyric acid (butyrate), hsa-mir-30d-5p–GPX3–butyric acid (butyrate), and hsa-mir-22-3p–ACADM–octanoylcarnitine (octanoylcarnit.). The hsa-mir-122-5p–IL6–bilirubin interaction network was formed by manually joining hsa-mir-122-5p–IL6 interaction with IL6–bilirubin interaction through protein IL6 that acts as a common interactor for hsa-mir-122-5p and bilirubin. The hsa-mir-122-5p–GSTM2–butyric acid (butyrate) interaction network was formed by manually combining hsa-mir-122-5p–GSTM2 interaction with GSTM2–butyric acid (butyrate) interaction through GSTM2 that act as common interactor for hsa-mir-122-5p and butyric acid (butyrate). Similarly, the hsa-mir-30c-5p–GPX3–butyric acid (butyrate) interaction network was formed by manually joining the hsa-mir-30c-5p–GPX3 interaction with the GPX3–butyric acid (butyrate) interaction through GPX3 that acts as a common interactor for hsa-mir-30c-5p and butyric acid (butyrate). Furthermore, the hsa-mir-30d-5p–GPX3–butyric acid (butyrate) interaction network is formed by manually combining the hsa-mir-30d-5p–GPX3 interaction with the GPX3–butyric acid (butyrate) interaction through GPX3 that acts as a common interactor for hsa-mir-30d-5p and butyric acid (butyrate). Furthermore, the hsa-mir-22-3p–ACADM–octanoylcarnitine (octanoylcarnit.) interaction network is formed by manually combining the hsa-mir-22-3p–ACADM interaction with ACADM–octanoylcarnitine (octanoylcarnit.) interaction through ACADM that acts as a common interactor for hsa-mir-22-3p and octanoylcarnitine (octanoylcarnit.). Notably, hsa-mir-122-5p–IL6 interaction and hsa-mir-122-5p–GSTM2 interaction were identified by TarBase and experiment such as microarrays. Furthermore, hsa-mir-30c-5p–GPX3 interaction, hsa-mir-30d-5p–GPX3 interaction, and hsa-mir-22-3p–ACADM interaction were identified by TarBase and experiment such as HITS-CLIP. Furthermore, IL6–bilirubin interaction, GSTM2–butyric acid (butyrate) interaction, GPX3–butyric acid (butyrate) interaction, and ACADM–octanoylcarnitine (octanoylcarnit.) interaction were identified with high confidence scores (≥0.7 or 70%).

In addition, IL6, GSTM2, GPX3, and ACADM form protein–protein and protein–metabolite interaction networks at high confidence scores (≥0.7 or 70%). Furthermore, bilirubin, butyric acid (butyrate), and octanoylcarnitine (octanoylcarnit.) form metabolite–metabolite and metabolite–protein interaction networks at high confidence scores (≥0.7 or 70%).

Interestingly, the expressions of hsa-mir-122-5p, IL6, FGL1, ACADM, LEP, INS, CASP1, NLRP3, ACAT1, TNF, ELAVL1, IGFBP7, ADAM9, GDF15, ACADVL, ECH1, octanoylcarnitine (octanoylcarnit.), isoleucine, leucine, L-valine, 7-keto-8-aminopelargonic acid (7-keto-8-amino.), creatinine, decanoylcarnitine (decanoylcarnit.), and hexanoylcarnitine (hexanoylcarnit.) were found to be upregulated in DCM, while those of hsa-mir-30c-5p, hsa-mir-30d-5p, hsa-mir-22-3p, GSTM2, GPX3, GSTM3, ADIPOQ, HADH, PRDX2, PRDX1, SERPINA1, A2M, PRDX6, APOA1, APCS, NPPA, bilirubin, butyric acid (butyrate), alanine, glutamine, and cytidine triphosphate (ara-CTP) were found to be downregulated in DCM.

We proposed miRNA–protein–metabolite interaction networks along with their intra- and inter-connected protein–protein, metabolite–metabolite, and protein–metabolite interaction networks formed by miRNA, protein, and metabolite fingerprints such as hsa-mir-122-5p, hsa-mir-30c-5p, hsa-mir-30d-5p, hsa-mir-22-3p, IL6, GSTM2, GPX3, ACADM, GSTM3, LEP, ADIPOQ, INS, CASP1, NLRP3, HADH, ACAT1, PRDX2, PRDX1, TNF, ELAVL1, SERPINA1, A2M, IGFBP7, PRDX6, APOA1, APCS, NPPA, ADAM9, GDF15, ACADVL, FGL1, ECH1, bilirubin, butyric acid (butyrate), octanoylcarnitine (octanoylcarnit.), isoleucine, leucine, alanine, glutamine, L-valine, cytidine triphosphate (ara-CTP), 7-keto-8-aminopelargonic acid (7-keto-8-amino.), creatinine, decanoylcarnitine (decanoylcarnit.), and hexanoylcarnitine (hexanoylcarnit.) may form the key players and the regulatory networks involved in the pathogenesis of diabetic cardiomyopathy (DCM).

Thus, the miRNA–protein–metabolite interactions along with their intra- and inter-connected protein–protein, metabolite–metabolite and protein–metabolite interaction networks may help select the key players and the regulatory networks involved in the pathogenesis of DCM. Additionally, they may also act as promising biomarkers of DCM and also serve as potential targets for DCM therapeutics. Furthermore, it can elucidate the new strategy for cardiovascular prevention in the case of cardiovascular–kidney–metabolic syndrome.

Notably, the interaction networks formed by miRNA, protein, and metabolite fingerprints involved in the early stage of DCM such as hsa-mir-122-5p, IL6, FGL1, LEP, ADIPOQ, INS, TNF, IGFBP7, GDF15, GPX3, NPPA, bilirubin, butyric acid (butyrate), and creatinine are the potential biomarkers for the early stage of DCM and also may be the therapeutic targets for the early stage of DCM.

This is the first study of the construction of miRNA–protein–metabolite interactomes in DCM providing insights into its pathogenesis, to the best of our knowledge. The experimental studies including *in vivo* and *in vitro* studies will be done in the future to investigate miRNA–protein–metabolite interactions such as hsa-mir-122-5p–IL6–bilirubin, hsa-mir-122-5p–GSTM2–butyric acid (butyrate), hsa-mir-30c-5p–GPX3 - butyric acid (butyrate), hsa-mir-30d-5p–GPX3–butyric acid (butyrate), and hsa-mir-22-3p–ACADM–octanoylcarnitine (octanoylcarnit.) interactions and other protein–protein, metabolite–metabolite, and protein–metabolite interactions in DCM.

Furthermore, in the future, the upregulated activity of forkhead box O1 transcription factor (FoxO1) in DCM as studied from preclinical models (Shafaati and Gopal, 2024) would need further evaluation for biomarker and therapeutic targets in the clinical studies of DCM. In addition, the role of perturbations in cardiac substrate metabolism inclusive of epigenetic alterations (Heather et al., 2024), role of ferroptosis including lipid peroxidation (Zhao et al., 2023; Cai et al., 2024), and the role of other modes of cell death like cuproptosis, autophagy, and others (Cai et al., 2024; Xuan and Zhang, 2023) are needed to be evaluated in the clinical samples of DCM.

## Data Availability

The datasets presented in this study can be found in online repositories. The names of the repository/repositories and accession number(s) can be found in the article/Supplementary Material.

## Abbreviations

DCM, diabetic cardiomyopathy; miRNAs, microRNAs; AGEs, advanced glycation end-products; T2DM, type 2 diabetes mellitus; NGR, non-diabetic subjects; PRDX2, peroxiredoxin 2; HADH, 3-hydroxyacyl-CoA dehydrogenase; ELAVL1, ELAV-like RNA-binding protein 1; SERPINA1, serpin family A member 1; ACADVL, acyl-CoA dehydrogenase very long chain; NPPA, natriuretic peptides A; TNNT2, troponin T, cardiac muscle, GSTM2, glutathione S-transferase Mu 2; ACAT1, acetyl-CoA acetyltransferase 1; FGL-1, fibrinogen-like protein 1; STITCH, Search Tool for Interacting Chemicals.
